# The recent relationship between ultraviolet-B radiation and biotic resistance in plants: a novel non-chemical strategy for managing biotic stresses

**DOI:** 10.1080/15592324.2023.2191463

**Published:** 2023-03-19

**Authors:** Gideon Sadikiel Mmbando

**Affiliations:** Department of Biology, College of Natural and Mathematical Sciences, University of Dodoma (UDOM), Dodoma, Tanzania

**Keywords:** UVB radiation, biotic stress, disease resistance, secondary metabolites, multiple resistance, flavonoids

## Abstract

Ultraviolet-B radiation (UVB; 280–315 nm) is a significant environmental factor that alters plant development, changes interactions between species, and reduces the prevalence of pests and diseases. While UVB radiation has negative effects on plant growth and performance at higher doses, at lower and ambient doses, UVB radiation acts as a non-chemical method for managing biotic stresses by having positive effects on disease resistance and genes that protect plants from pests. Understanding the recent relationship between UVB radiation and plants’ biotic stresses is crucial for the development of crops that are resistant to UVB and biotic stresses. However, little is known about the recent interactions between UVB radiation and biotic stresses in plants. This review discusses the most recent connections between UVB radiation and biotic stresses in crops, including how UVB radiation affects a plant’s resistance to disease and pests. The interaction of UVB radiation with pathogens and herbivores has been the subject of the most extensive research of these. This review also discusses additional potential strategies for conferring multiple UVB-biotic stress resistance in crop plants, such as controlling growth inhibition, miRNA 396 and 398 modulations, and MAP kinase. This study provides crucial knowledge and methods for scientists looking to develop multiple resistant crops that will improve global food security.

## 1. Background

Since photosynthesis depends on sunlight, plants are constantly exposed to ultraviolet-B radiation (UVB; 280–315 nm) from the sun. UVB prevents photosynthesis and protein synthesis, which reduces growth and productivity in plants. According to their wavelength, solar UV light falls into one of three categories: UVC (200–280 nm), UVB (280–315 nm), and UVA (315–400 nm)^1^. Even though UVB radiation makes up only 1.5% of all radiation^2,3^, it is of utmost importance because it has damaging effects on DNA that have an impact on a plant’s growth and development. UVB damages the photosynthesis machinery and DNA of nuclear, chloroplasts, and mitochondria^4–7^, increases the production of phenolic compounds^8^, and increases oxidative pressures in plants^9^. A decrease in PSII proteins D1 and D2 in photosystem II (PSII) can cause UVB radiation to have an impact on the primary photosynthesis machinery^10^. Cyclobutane pyrimidine dimers (CPDs) and (6–4) pyrimidinone photoproducts (6–4 PPs) are the two main UVB-induced DNA lesions; CPDs account for 75% of the lesions and 6–4 PPs for the remaining portion^11,12^. The performance and productivity of crops may be affected by these DNA lesions, especially those grown in tropical regions where UVB radiation levels are high.

UVB light can stimulate the UV-B photoreceptor UVR8 (UV-B RESISTANCE 8), which alters the expression of several genes important for UV-light stress acclimation including DNA repair enzymes^13^. Plants have developed several defense mechanisms to prevent UVB-induced lesioning, including DNA repair mechanisms by nucleotide excision repair (NER) and photoreactivation by the photolyase enzymes. The enzyme photolyases mediate photoreactivation (photorepair), which is the most efficient and practical method for plants to repair DNA lesions caused by UVB radiation^14,15^. The activity of the CPD photolyase enzyme has a significant impact on the UVB sensitivity of crops like rice^6,7,16,17^. In some studies^18–20^, transgenic rice plants with improved CPD photolyase activity due to overexpressing the CPD photolyase gene have higher UVB resistance mechanisms than their parental lines (PLs) plants. Additionally, mutation of even a single amino acid in the CPD photolyase gene led to highly UVB-sensitive phenotypes^6,7^. According to these studies, CPD photolyase activity is beneficial for plants to establish UVB resistance mechanisms. In other mechanisms, chloroplasts may be moved into various positions to reduce the amount of high light and UVB they absorb, restricting PSII damage^21–23^. The ability of plant phenolics, such as flavonoids and phenolic acids, to act as UVB screens and reduce the amount of radiation that reaches the mesophyll cell has also been demonstrated to lessen the oxidative stress brought on by UVB radiation^24,25^. UV light has significant effects on a plant’s general growth and development, including the direct harm it causes to plant pathogens and the improvement of disease resistance^26,27^. For instance, Mmbando et al. recently showed how UVB radiation enhanced resistance to the UVB-sensitive line A-S of the *Magnaporthe oryzae*, a rice blast fungus^28^. Their findings suggested that there may be a cross-tolerance between how plants react to UVB radiation and other biotic stresses like fungi. Knowing how these two factors interact makes it more likely to create crops that can withstand UVB and other biotic stresses. However, there haven’t been a lot of recent studies on how UVB radiation interacts with other biotic stresses.

**Figure 1.**
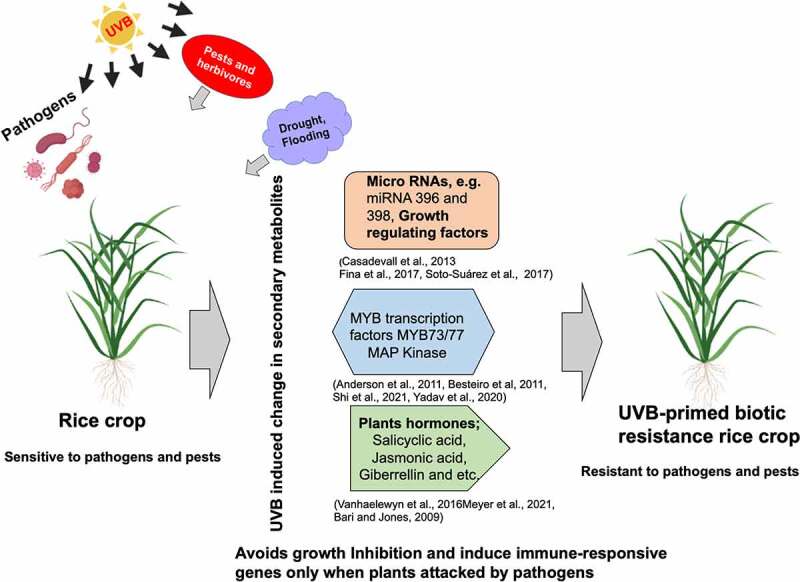
The current strategies for generating UVB and biotic resistance crops.

The need for the development of UVB-resistant crops is urged by the significantly higher level of UVB radiation penetration in recent years due to ozone layer depletion as a result of increased contamination of the atmosphere by pollutants like chlorofluorocarbons^29–31^. According to Germ et al., this increase in UVB radiation can also change community dynamics and ecosystem processes via many pathways, including species competition, carbon budgets, and biogeochemical cycles^32^. It can also have an impact on a plant’s defense-related secondary metabolite biosynthesis^33^. Surprisingly, tropical regions with higher levels of UVB radiation, such as Bengal and Africa, have domesticated high UVB-sensitive cultivars, such as African rice (*O. glaberrima*) and Surjamkhi (*O. sativa* L. ssp. indica)^6,7,34^. The maintenance of immune defenses as a result of an immune challenge and defense against high UVB-induced DNA damage due to ozone layer depletion are both energy-intensive processes^35^. The trade-off between competing demands for limited energy resources could lead to less than ideal performance in one or both processes^35^. For instance, plants in tropical areas with high biotic stresses might decide to redirect their energy into immunity for fending off biotic stresses, leaving them without any energy for repairing UVB damage. As an alternative, the high sensitivity to UVB sensitivity may also lead to a compromise in the allocation of carbon in order to repair UVB stress while leaving less carbon for pests and pathogens that feed on plants, hence decrease disease severity. Because plants can tolerate some accumulation of UV damage such as CPDs, it has been hypothesized that high UVB sensitivity traits may be useful in protecting these crops from other biotic stresses like pathogens through trade-off mechanisms, even though the reasons for cultivating high UVB-sensitive cultivars in tropical regions are not yet known^34,36^. This idea was recently supported by a study by Mmbando et al., which showed that transgenic lines and cultivars with high UVB sensitivity and low UVB tolerance improved their resistance mechanisms to *M. oryzae*^28^. Plants have developed a network of interactions as a result of being subjected to multiple stresses at once, including crosstalk with different signals that determine how well stress tolerance genes function^37^. One stress promotes cross-tolerance to other stresses (Figure 1). In elements similar to those brought on by pathogen attack, UVB radiation has been shown to act through a downstream pathway^38,39^. For instance, the increased level of sinapates (sinapoyl malate and sinapoyl glucose) in Arabidopsis following UVB irradiation was responsible for the plant’s increased resistance to *Botrytis cinerea* ^40,41^. Additionally, earlier research has suggested that excluding UVB from natural sunlight increases the severity of the powdery mildew disease^42^. As a result, it is thought that the ambient UVB level affected biological processes such as morphological change, herbivores, and microbial pathogen resistance, and change in biochemical composition^39,43,44^. Cross-tolerance knowledge and an understanding of the recent relationships between UVB radiation and other biotic stresses may open doors for the creation of multiple resistant crops for raising yield productivity in the face of shifting global climatic conditions. There is, however, a paucity of current data on the interaction between UVB radiation and other biotic stresses.

UVB radiation and biotic stresses both have an impact on plants grown in a typical environment. The induction of defense-responsive genes, hormones, and secondary metabolites with biotic resistance properties is made possible by targeting micro-RNA 396 and 398 as well as growth-regulating and transcription factors, which control the downstream targets of UVB-induced growth retardation. This will produce plants that are UVB-primed biotic resistance, with enhanced biotic resistance mechanisms.

Plants may experience direct or indirect effects from UVB radiation, depending on the dose^45^. For instance, the employ of UV-screening filters had direct effects on the Blister Blight disease of *Camellia sinensis* (tea), which is caused by the fungus *Exobasidium vexans*. Such effects showed that the UVB components of solar radiation reduced the germination rates and survival of the affected plants, which ultimately resulted in a decrease in the disease^46^. Additionally, according to Kunz et al., UV exposure to plants could activate defense mechanisms without the need for Avr recognition by R protein^47,48^. Moreover, it has been demonstrated that UVB activates genes such as ß-1, 3-glucanase, chalcone synthase, chitinase, lipoxygenase, stilbene synthase, phenylalanine ammonia-lyase (PAL), and PR proteins that are essential for pathogen resistance in plants^49–57^. Besides this, it has been demonstrated that UVB acts through downstream signaling pathways containing elements related to those for pathogen defense, such as mitogen-activated protein kinases^58^, calcium^59^, jasmonic acid (JA), salicylic acid, and ethylene^39^, ROS, and nitric oxide^60^; and Tomatoes’ ability to resist herbivores can be regulated by UVB radiation exposure by signaling through JA^61^. Furthermore, UV-B strengthens plant defenses against numerous plant species as well as lepidopteran insects through a mechanism that depends on JA^62^. All of these findings indicated that UVB radiation has a significant impact on biotic stress, particularly on pathogen resistance. It has been hypothesized that domesticating high UVB-sensitive cultivars in tropical regions with high UVB stress helps those plants survive other biotic stresses^36^. Future research will focus heavily on figuring out the molecular mechanisms underlying the trade-off between pathogen resistance and UVB sensitivity. We need to figure out the best way to create multiple resistant crops because of the rise in UVB radiation brought on by ozone layer loss and the appearance of new plant pathogens as a result of climate change and global warming. This is crucial for agricultural production, especially in tropical areas where crops are under intense pressure from UVB rays and biotic stresses at the same time. Understanding the key players in the relationship between UVB radiation stress and biotic stresses will be crucial to provide information on developing crops that are resistant to both UVB and biotic stresses because it has been demonstrated that UVB radiation influences various biotic stresses. Consequently, UVB radiation could be used in agriculture as a non-chemical and best approach to control biotic plant stresses, thereby reducing the need for pesticides that are bad for human health and the environment. Additionally, supplementing UVB radiation as a “treatment” has no residual effects, in contrast to the long-lasting effects of chemical plant growth regulators that take effect once the plant absorbs them^1^. However, there is not much information on the recent interaction between UVB radiation due to ozone layer depletion and biotic stresses.

The most recent studies on the connection between UVB radiation and resistance to biotic stresses were the main focus of this review. Given that plants are currently under numerous stresses as a result of climate change, the knowledge presented here will be useful in identifying strategies for developing multiple resistance crops. The results of this study will be used to create non-chemical, environmentally responsible strategies for biotic stress management. The relationship between biotic stress and UVB provided here might be improved. The relationship between UVB and biotic stress that has been established here may enhance food security, particularly in tropical regions where pathogens are a major source of biotic stress and UVB exposure is high.

## 2. UVB radiation and plant disease resistance

For improving the disease resistance mechanism in plants, various UVB doses have been used in various studies, and the daily doses have been shown to vary between 0.2 and 32 kJ^−2 40^. Finding a specific dose that will activate a defense response while not harming the crop is very difficult. For instance, sunflower cotyledons can withstand daily doses of 30 kJ m^−2^ while broccoli florets can show signs of phototoxicity at a daily dose of 2.2 kJ m^−2 63^. Therefore, scientists need to select an appropriate UVB dose that is specific to plants; otherwise, different pathways might be activated that either causes stress or light acclimatization reactions. However, plants’ perception of UVB radiation triggers the production of several metabolites, which are essential for adapting to UVB radiation. This acclimatization could be accomplished by either increasing antioxidant capacity or by directly applying sunscreen to reduce the absorption of excessive UVB radiation^64^. Phenolic compounds, alkaloids, glucosinolates, and some hormones like JA and salicylic acids (SA) are examples of specialized metabolites induced by UVB radiation that may strengthen plants’ defenses^40^; there is also evidence that secondary metabolites contribute to UVB-induced pathogen resistance^41^.

Numerous phenolic substances, such as nobiletin, flavones, quercetin, and genistein, exhibit antifungal properties by altering the permeability of the microbe cell wall, either by directly inhibiting fungal growth in the apoplast or by interacting with fungal membrane proteins^65,66^. This suggested that the relationship between UVB and improving disease resistance in UVB-treated plants is through the upregulation of these metabolites by UVB radiation. For instance, after being exposed to UVB light, the increased levels of sinapates (sinapoyl glucose and sinapoyl malate), which are mediated by UVR8, increase Arabidopsis’ resistance to the fungus *Botrytis cinerea* ^40,41^. Additionally, UVB led to increased amounts of isoflavone glycosides and enhanced resistance to herbivores in a variety of soybean cultivars; the only cultivar that was nonresponsive to UVB radiation and without strong isoflavone activation displayed low resistance to the pests^67^. A recent study by Fardhani et al. showed that only in the cucumber leaves (*Cucumis sativus* L.) that had been exposed to UVB radiation did the expression of defense-related genes increase, reducing the severity of powder mildew^68^. Additionally, UVB radiation can activate hormones that give plants their disease resistance. For example, numerous studies have demonstrated that exposure to UVB light has an impact on the synthesis of plant hormones like JA and SA. While SA is known to be involved in defense responses toward biotrophic organisms, JA is known to be involved in defense responses against necrotrophic pathogens and herbivores^69,70^. Although altering the experimental conditions may yield contradictory results for JA accumulations in response to UVB light^40^, Arabidopsis plants exposed to UVB radiation showed an upregulation of genes associated with defense and SA without inducing UVB stress, downregulation of genes implying photosynthesis, variation in chlorophyll content, or peroxide and hydrogen peroxide accumulation^71^. Similar to this, another study showed that tomato plants exposed to UVB light for 11 days did not show any signs of UVB stress but did significantly accumulate salicylic acid beta-glucoside (SAG) and SA^72^. These studies suggested that the improvement in plant resistance to biotic stresses following exposure to UVB radiation is modulated by hormones, particularly JA and SA.

Many plants can produce pathogenesis-related (PR) proteins when exposed to UVB radiation^73^. For instance, UVB causes the activation of various pathogenesis-related substances, including the PR protein OSMOTIN-LIKE PROTEIN (OLPs), which has antifungal properties in rice^40^. UVB radiation stimulates ß-1.3-glucanase (PR-2) in strawberries and rice, as well as in response to *Magnaporthe oryzae*, a rice blast fungus^74,75^. Similar to this, UVB radiation activates genes related to bacterial and fungal pathogens, including ß-1,3-glucanase and PR-4, which are responsive to JA, and PR-2, SA, and PR-1, which are responsive to SA^76^. Recent research by Mmbando et al. have shown that pretreatment with low doses of UVB reduces the length and number of multicellular infectious hyphae in lesions, which in turn lessens the severity of *M. oryzae* in UVB-sensitive lines^28^. Curiously, UVB pretreatment also causes several PR proteins and defense genes to become active in uninoculated plants. According to those findings, UVB radiation may strengthen plants’ defenses even before they become infected by pathogens by activating some defense mechanisms, including PR proteins. A plant may mistake UVB radiation for pathogens and prepare its defense mechanism so that it is ready to defend against actual pathogens when they come into contact with the plant. As the most promising method of using UVB radiation as a non-chemical tool to control biotic stress, this UVB priming phenomenon will need more attention. Finding the right UVB dosage for a specific species is crucial because different plant species have varying rates of tolerance to UVB sensitivity, even though both high and low UVB dosages can increase a plant’s resistance to pathogens and pests. The best method for increasing UVB and pathogen resistance in crops may therefore be to target these metabolites activated by UVB radiation while also managing UVB-induced growth inhibition effects.

## 3. UVB radiation and herbivore and pests resistance

UVB radiation has been implicated in influencing the performance, survival, and behavior of herbivores and their adversaries, even though high levels may hurt plant performance^77^. The reduction in performance and/or preference of herbivorous antibodies in different plant species were shown in UVB supplemental and exclusion studies^41–76–78–80^. Depending on the types of plants and herbivores, UVB interactions have a wide range of effects on biotic interactions. For instance, both insect herbivores and exposure to solar UVB trigger the partially overlapping phenolic profiles and gene expression in *Nicotiana attenuata* and *Nicotiana longiflora* plants^81^. Herbivorous arthropods’ behaviors and preferences can change as a result of UVB-mediated changes in plant architecture, chemistry, and/or physiology, particularly when plant defenses are strengthened^82^. It has been suggested that UVB-induced cell wall augmentation and/or increased production of UVB-protective secondary metabolites may affect how herbivorous arthropods develop in plants^76,78^. There is an overlap between herbivore attacks and plant responses to UVB that may have the same impact on plant defenses, even though an antagonistic relationship between these responses may also happen and reduce plant defenses^82^. The activation of specific diterpene glycosides, for example, is mediated by UVB and is a key component of *N. attenuata* defenses against the mirid *Tupiocoris notatus*. As demonstrated by Mewis et al. on the mediation of stimulation of two different plant defense-related metabolites, glucosinolates, and flavonoids, in broccoli (*Brassica oleracea*) sprouts, UVB radiation can adjust the production of various plant chemicals that differ in their influence on plants’ resistance^76^. The UVB-mediated adjustment of activated plant defenses can be closely related to how much a plant’s chemical alteration activated by UVB contributes to antiherbivore properties^82^.

The thickness of leaf trichomes, which are hairy epidermal structures that shield plants from abiotic stresses like extreme light intensity and UV radiation as well as herbivory resistance, has been shown to increase in response to UVB radiation^83–85^. Herbivore resistance is significantly mediated by phytohormones. For instance, increased plant resistance to piercing-sucking, some phloem-feeding arthropods, and leaf-chewing is associated with stimulation of JA signaling^86^. Additionally, pre- and post-herbivory constitutive (i.e., pre-herbivory) jasmonate levels can be increased by UVB radiation, which increases chemical defenses^62,87^. These studies suggested that the influence of UVB on various phytohormones, including cytokinin, auxin, and gibberellins, which altered the leaf chemistry, is responsible for the improved resistance to herbivores^82^. Before using UVB radiation as a stimulus in crop production, these responses must be clarified because while UVB radiation increases plant resistance to herbivores, it can also inhibit growth by inhibiting auxin signaling^88,89^. Besides, it has been proposed that UVB may have strengthened broccoli plants’ resistance to cabbage aphids by increasing the concentration of flavonoids^79^. Furthermore, other studies have demonstrated that UVB radiation can change a plant’s susceptibility to insect chewers. For instance, in contrast to reduced UVB conditions, there was little damage to soybean plants caused by chewing herbivores under ambient UVB radiation in field conditions^80^. UVB radiation also helped white clover (*Trifolium repens*) become more resistant to *Spodoptera litura* ^90^. Since the JA pathway was shown to increase maize and rice resistance to insects through UVB, some evidence suggests that the JA pathway is involved in the resistance, even though the mechanisms by which UVB affects plant resistance to herbivores are not yet fully understood^62^.

According to Kuhlmann and Müller., growth under UV irradiation changed the plants’ allure to herbivorous insects like aphids, thrips, and whiteflies^91^. Thus, UVB radiation has emerged as a light signal with potential applications to increase crop yield and strengthen plant defense against insect pests without the use of pesticides^80^. In other words, UVB can be viewed as a “eustress,” a type of stress that has positive effects on plant health and facilitates adaptation to extremely demanding stressful situations^92^. In addition to biotic stresses like above-ground arthropod herbivores, UVB has been shown to confer cross-tolerance to abiotic stresses like high temperatures, light stress, and drought^61,87,93,94^. The UVB effect on the synthesis of secondary metabolites may also be influenced by other environmental factors. For instance, Arabidopsis plants grown outdoors under conditions of moderate temperature and low daily light integrals did not demonstrate a significant effect of UVB on the production of phenolics^95^. Accordingly, variations in climatic conditions, particularly in outdoor and experimental growth chamber experiments, may affect the UVB response to secondary metabolite production, which affects resistance to pathogens and pests. This would account for the wide range of results in various studies on the response to UVB on disease and pests in plants. Because some of the activated specialized metabolites that ward off insects and microorganisms may also be unpleasant for human consumption, such as bitter taste, the beneficial effects of UV on plant resistance must be carefully considered^1^.

## 4. Possible strategies to develop both UVB and pathogen resistance in crops

UVB radiation that reaches the earth’s surface increases as the ozone layer thins^29,30^. Similar climatic and global warming changes, such as an increase in UVB, may affect plants’ production of defense-related secondary metabolites, which may increase the frequency and severity of pathogens and pests in some areas^96,97^. Recently, Mmbando et al. showed a connection between high UVB sensitivity and disease resistance mechanisms in *M. oryzae*, indicating the significance of UVB radiation for enhancing disease resistance mechanisms^28^. To increase crop productivity in the current climate, methods for breeding crop plants with multiple resistances will be of utmost importance. It may be possible to create crops that are resistant to pathogens and UVB stresses by investigating the trade-off between disease resistance and UVB sensitivity in crops^36^. However, there is little information available regarding potential methods to produce crops that are resilient to biotic and UVB stresses. This could hinder the development of the green revolution, especially in tropical regions with high levels of UVB radiation and pathogen stress, where breeders and biotechnologists might find it difficult to create multiple resistant crop plants. Thus, a method is required that can balance the trade-off mechanism by preventing UVB’s effects on plant growth inhibition^6,7^, while allowing the activation of the plant’s defenses without sacrificing growth. Here are some potential strategies for creating crops that are resilient to UVB radiation as well as pathogens, pests, and disease stress.

### 4.1 controlling the effects of UVB on plant growth inhibition

The number of tillers, plant height, and cell proliferation in proliferating cells are all suppressed by UVB radiation^6,7,98^. By boosting CPD photolyase activity, plants may be able to withstand this inhibition. However, it has recently been demonstrated that high CPD photolyase activity reduces resistance to *M. oryzae*; Mmbando et al. suggested that a moderate level of CPDs may be required to activate the disease resistance mechanism in plants^28^. Thus, when examining the advantages of UVB radiation on pathogen resistance or even developing UVB-pathogen-resistant plants, controlling the downstream targets of UVB-induced growth inhibition effects in plants is crucial. Growth regulating factors (GRFs), which have been linked to controlling cell proliferation in response to UVB radiation^99,100^, maybe the downstream targets for UVB-induced growth inhibition effects on plants. Examples of GRFs include GRF1, GRF2, and GRF3 in Arabidopsis^99^ and possibly GRF1, GRF2, GRF14, and GRF15 in maize^100^. Using gene editing methods like clustered regularly interspaced palindromic repeats (CRISPR-Cas9) may be able to control the miRNAs or GRFs responsible for preventing cell proliferation, preventing the negative effects of UVB radiation while allowing the positive effects of priming disease and pathogen resistance. Although different GRFs may serve different purposes in different species, manipulating the miRNAs or GRFs^99,100^ that are responsible for preventing cell proliferation using gene editing methods like CRISPR-Cas9^101^ may prevent the negative effects of UVB radiation on plant growth while favorably influencing the development of disease and pathogen resistance mechanisms in these plants. Another strategy could be to inhibit auxin signaling, which would combine the auxin and UVB signaling pathways. For example, the MYB transcription factors MYB73/77 control lateral root growth by interacting with UVB in roots when UVB is present^102^. Therefore, the MYB73/77 transcription factors may be responsible for root growth inhibition in plants exposed to UVB radiation. By adjusting these transcription factors, it may be possible to lessen the effects of growth inhibition while also enhancing resistance mechanisms. This strategy aims to prevent a plant’s growth or cell division from being inhibited while allowing the priming of disease and pathogen resistance effects on plants through UVB-induced changes to plant chemistry.

### 4.2 modulating miRNA 396 and 398

When exposed to a low dose of UVB treatment (0.4 Wm^−2^), high UVB-sensitive cultivars showed greater disease resistance against *M. oryzae*, indicating that UVB-induced accumulation of CPDs is required for the induction of disease resistance mechanisms^28^. However, the high concentration of CPD accumulations will prevent plant growth at high UVB radiation doses (1.2 Wm^−2^)^7,18,20,103^. Therefore, developing multiple resistance plants will require molecular methods of regulating the level of CPDs or controlling the downstream target of CPDs, perhaps through miRNA. According to Jia et al., findings from a filter assay, UVB radiation raised the levels of the miRNAs miR156, miR160, miR165/166, miR167, and miR398 in *P. tremula* ^104^. Small non-coding RNAs called microRNAsplays several biological roles, such as regulating growth in many plants and responding to abiotic and biotic stresses, and stress responses^105,106^. MicroRNAs are essential for the UVB-induced aggregation of bioactive plant components^107^. For instance, UVB treatment in Arabidopsis has been shown to up-regulate 21 microRNA genes in 11 microRNA families^108^. In addition, it has been demonstrated that the microRNA 396‘s suppression of GRFs prevents UVB radiation from causing cell proliferation in Arabidopsis leaves^99^. By modifying innate immunity without affecting growth, miRNA396 has a significant impact on plants’ immunity^109^. As a result, miRNA396 may be the link between plant immunity and UVB radiation. Using molecular methods to regulate its expression level or downstream targets GRFs could be a useful strategy for creating UVB-resistant primed disease-resistance plants. Other miRNAs, including miRNA398, have been demonstrated to link the plant stress regulatory network to responses in plants to water shortage, abscisic acid, copper, phosphate, ultraviolet stress, bacterial infections, and higher sucrose^110^. Therefore, manipulating miRNAs 396 and 398 may hold the key to creating crops resistant to UVB stress and other biotic stresses. However, more research is required to comprehend other miRNAs that interact with disease metabolism and UVB.

### 4.3 Modification of MAP kinase

It has been demonstrated that MAPK PHOSPHATASE 1 (MKP1) contributes to UV resistance when UVR8-regulated UVB acclimation cannot withstand UVB stress^111,112;^ additionally, the Arabidopsis MKP1 fault mutant mkp1 is more vulnerable to UVB than the wild-type^113^ indicating the significance of this system in UVB protection. MPK3 and MPK6 are downstream targets of MKP1 that UVB stress is known to activate; MKP1 functions as a contra-regulator of MPK3 and MP6 activities in plant resilience to UVB and salt stresses, as well as in the bacterial pathogen *Pseudomonas syringae* ^111,112,114^. Controlling the activity of MKP1 or its downstream targets MPK3 and MPK6 may allow the possibility of developing UVB and pathogen or disease-resistant crops because MKP1 is involved in improving resistance to both biotic and abiotic stresses. Hormone synthesis and signaling can be affected by mitogen-activated protein kinase (MAPK). For instance, it has recently been shown that MPK3 and MPK6 are essential for SA priming in Arabidopsis^115^. Multiple resistant plants can be created by combining all of these tactics, including the hormonal pathways for JA and SA. However, more research is required to determine the ideal UVB dosage for inducing defense mechanisms in a variety of cultivars without affecting growth. Additionally, this is a recent and active research area that calls for more confirmation experimentation. Even so, the study of multiple resistance to UVB and biotic stress is a relatively unexplored but highly intriguing and promising field of study because it presents numerous opportunities for breeding plants that are resilient to multiple stresses. This will be crucial in increasing food production to feed densely populated world, especially in tropical regions that experience multiple stresses instantly.

## 5. Conclusion

The review’s main focus is on the most recent connection between UVB radiation and biotic stress resistance. Recent studies^28,40,116^ have found that UVB radiation affects plant chemistry in a way that makes them more resilient to biotic stresses like pathogens, and pests. Understanding the relationship between these two factors may lead to the development of crops that are resistant to both UVB radiation and biotic stress. Recent research has revealed that low UVB resistance and CPD photolyase activity are required for improving biotic resistance to *M. oryzae* in rice^28^, although most studies have focused on increasing UVB resistance by enhancing CPD photolyase activity^18–20^. Plants may require a sufficient number of CPDs to activate the UVB-mediated biotic resistance mechanism. Thus, the knowledge provided here will expand our understanding of the relationship between UVB sensitivity and disease resistance mechanisms in plants. The molecular mechanism underlying this relationship should be clarified in future studies as it will aid in the development of UVB-resistance primed plants. However, because most laboratory conditions use artificial UVB lighting conditions with unnaturally high or low UVB fluxes and doses, it is necessary to test whether the anticipation made based on controlled laboratory conditions will work well under natural conditions in the field. Field experiments should be carried out before the commercialization of various crop varieties. This study revealed the most recent link between UVB radiation and biotic stress resistance, which provides crucial information for creating multiple resistant crops. The development of multiple resistance crops will offer ways to increase crop productivity, which can end hunger and poverty in the world because many crops are used as both food and for earning income.
